# Cross-cultural adaption and validation of the German version of the Mini-BESTest in individuals after stroke: an observational study

**DOI:** 10.1186/s42466-020-00078-w

**Published:** 2020-10-01

**Authors:** Elena Cramer, Franziska Weber, Gilian Faro, Michael Klein, Dennis Willeke, Thomas Hering, Dörte Zietz

**Affiliations:** 1 Department of Applied Health Sciences, Division of Physiotherapy, Hochschule für Gesundheit (University of Applied Sciences), Gesundheitscampus 6-8, 44801 Bochum, Germany; 2 Rehabilitation Center for Neurology, Neurosurgery and Neuropaediatrics, VAMED Klinik Hattingen GmbH, Am Hagen 20, 45527 Hattingen, Germany; 3 Rehabilitation Center for Neurology and Orthopaedics, Johanniter-Klinik am Rombergpark, Am Rombergpark 42, 44225 Dortmund, Germany; 4 Department of Applied Human Sciences, Hochschule Magdeburg-Stendal (University of Applied Sciences), Osterburger Str. 25, 39576 Stendal, Germany

**Keywords:** Postural control, Measurement instrument, Mini-BESTest, Validity, Stroke, Cross-cultural adaption

## Abstract

**Background:**

Postural control is a very important function in everyday life. However, assessing postural control with commonly used measurement instruments (MIs) is limited due to deficits in their psychometric properties. The Mini-Balance Evaluation Systems Test (Mini-BESTest) is a comprehensive and multidimensional MI for assessing postural control in persons with limited balance function, such as individuals after stroke. Despite the increasing use of the Mini-BESTest worldwide, no German version is available.

**Research question:**

Is the German version of the Mini-BESTest (GVMBT) comprehensible and valid for measuring postural control in individuals after stroke?

**Methods:**

The Mini-BESTest was translated and cross-culturally adapted, following established guidelines. It was pilot-tested with ten participants. This observational measurement and validation study was conducted at one point and included 50 participants with subacute and chronic stroke (mean age: 64.58 ± 13.34 years/ 34 men/ 16 women). Convergent validity was investigated using 1) the Berg Balance Scale (BBS) and 2) the Timed “Up & Go” (TUG). The MIs were evaluated for normal distribution with the calculation of skewness, kurtosis and Q-Q-Plots. Spearman correlation coefficients and Bland Altman analysis were used to examine the relationship between the MIs. The internal consistency was assessed using Cronbach’s alpha.

**Results:**

Comprehension of the GVMBT was confirmed. The GVMBT correlated significantly with the BBS (r_s_ = 0.93) and the TUG (r_s_ = − 0.85). Bland Altman analysis revealed low absolute differences. The GVMBT demonstrated no significant floor or ceiling effects and showed excellent internal consistency (Cronbach’s *α =* 0.90).

**Significance:**

The GVMBT has excellent validity and internal consistency. Due to this and its specific subcategories, the GVMBT is recommended for the use in research and clinical practice. Further psychometric properties should be evaluated.

## Introduction

Postural control is important in activities of daily living [1]. It involves the ability to control the position of the body for stability and orientation [2]. Postural stability, commonly referred to as balance, is the ability to control the center of mass in relation to the base of support [2]. Maintaining a task-specific relationship between body segments and between the body and the environment is defined as postural orientation [2]. Postural control forms the foundation for all movements against gravity, for selective movements and for functional tasks [3]. It depends on different subsystems, which have to cooperate with each other [2]. Impaired postural control has been associated with poorer functions in activities of everyday life, reduced ambulatory capacity, an increased risk of falling, limited social participation and it is one of the most common disabilities after stroke [1, 3]. Stroke is the second leading cause of death worldwide [4]. More than 80% of people who had first-time strokes, showed balance impairments resulting in limited sitting balance, standing balance or stepping balance [1, 3]. Hence, it is necessary to gain a clearer understanding of balance dysfunctions after stroke, in order to specify intervention programs.

In order to assess postural control, a standardized measurement instrument (MI) is important [5, 6]. MIs provide quantifiable and objective data to support clinical reasoning and are a crucial part of evidence-based practice [5, 7]. Many different MIs are used to assess impaired postural control, but there is a lack of a clinically useful reference standard [6]. The most commonly used MIs in German speaking countries are the Berg Balance Scale (BBS) [8, 9], the Timed “Up & Go” (TUG) [10, 11], the Functional Reach Test [12], the One Leg Standing Test [12] and the Dynamic Gait Index [13]. However, few MIs consider the different aspects underlying postural control. For instance, the BBS lacks important aspects of dynamic balance function, for example gait items or the ability to react to postural perturbations [14]. The TUG or the One Leg Standing Test, being single-task MIs, can only be used as descriptive tools [6, 11]. Moreover, the BBS, the Functional Reach Test and the One Leg Standing Test demonstrate significant floor and ceiling effects in measuring postural control in individuals after stroke [6, 9, 13].

Addressing the majority of these problems, the Balance Evaluation Systems Test (BESTest) has been developed [15]. The time-consuming administration of the BESTest (35 min) is a key barrier for clinical utility [14]. With Rasch and factorial analyses a shorter version of the BESTest has been developed, called Mini-BESTest [14]. This shorter version still encompasses almost all underlying components of postural control. It has already been tested in many different populations and shows robust psychometric properties [16]. The criterion validity has been primarily analysed with correlations between the Mini-BESTest and different other balance measures. The Mini-BESTest and the BBS demonstrated good to excellent correlations (Pearson’s r between 0.79–0.94 and Spearman’s p between 0.83–0.85) [16] and further studies revealed strong positive evidence for internal consistency (Cronbach’s α ranging from 0.89–0.96) [16]. So far, there is no study, reporting ceiling or floor effects of the Mini-BESTest [16]. With regard to these properties, the Mini-BESTest represents a reliable, valid and responsive MI. It has gained acceptance in clinical practice and research and has been translated and cross-culturally adapted into various languages [16].

To our knowledge, there is no German version of the Mini-BESTest (GVMBT) available, which has been formally translated. Accordingly, the objective of this study was to translate and cross-culturally adapt the Mini-BESTest into the German language following established guidelines of Beaton, Bombardier, Guillemien & Ferra [17] and to validate the GVMBT with individuals after stroke. We hypothesized that (1) the BBS and the TUG measure some aspects of postural control [16] and therefore, these MIs would reveal moderate to high correlations with the GVMBT. Moreover, (2) the GVMBT would measure postural control more precisely and accurately compared to the other used MIs due to high correlations and good agreement in the Bland Altman plots. Furthermore, it would show neither floor nor ceiling effects [16].

## Methods

This observational measurement and validation study was approved by the ethics committee of the “Hochschule für Gesundheit Bochum”. It was conducted at one point and followed the “STrengthening the Reporting of OBservational studies in Epidemiology (STROBE)” guidelines to standardize reporting [18].

### Translation process

According to the guideline suggested by Beaton et al. (2000), the original version of the Mini-BESTest was translated and cross-culturally adapted from May to August 2018 [17]. All six recommended stages of cross-cultural adaption were conducted; for instance, the translation of the original version into the German language and the back-translation by two independent persons, respectively. A committee of experts created a pre-final version. This pre-final version was pilot-tested with ten participants and linguistic expressions in the GVMBT were finalised. In correspondence with the developers of the original Mini-BESTest, an experts’ committee created a final German version.

### Study settings

Data acquisition took place at two neurorehabilitation centers in North Rhine-Westphalia, Germany, from October 2018 to January 2019. Two experienced physiotherapists assessed all participants. In order to guarantee standardized implementation, the physiotherapists watched the original training videos of the Mini-BESTest and practiced the handling of the MIs on healthy persons.

### Participants

All participants provided written informed consent. Inclusion criteria were: individuals (1) after stroke, (2) with hemiplegia, (3) able to walk at least six meters (walking aids allowed), (4) with sufficient cognitive abilities regarding verbal communication and understanding instructions in the German language, (5) aged ≥18 years and (6) physically able to perform different tasks for 1.5 h. Exclusion criteria were: individuals (1) with ataxia, (2) with deafness or blindness, (3) in clinical isolation, (4) with contraindications for mobilization, (5) with acute pain, (6) who were pregnant, (7) with symptoms of vertigo and (8) with other conditions influencing the balance ability for example polyneuropathy. Since the “COnsensus-based Standards for the selection of health Measurement INstruments (COSMIN)” guidelines do not recommend a specific sample size for validity analysis [19], the sample size was therefore oriented on similar validity studies [20–22]. Due to the wide range of different sample sizes reported in similar studies, sample size calculation for validity analysis was done a priori with an effect size of 0.5, an alpha error of 0.05 (two-tailed) and a power of 0.9 [23].

### Procedure & outcome measures

Initially, the National Institute Health Stroke Scale (NIHSS) was performed to describe the study population. The testing sequence (Mini-BESTest, TUG and BBS) was randomly assigned to the participants, avoiding bias because of fatigue [18]. The administration time of the Mini-BESTest was recorded for each participant. Additionally, each participant was asked about the comprehensibility of the test instructions. Blinding of the physiotherapists was not possible.

The Mini-BESTest is a 14-item balance test with four subcategories: ‘anticipatory control’, ‘reactive postural control’, ‘sensory orientation’ and ‘dynamic gait’. Each item is scored from 0 to 2. A higher score indicates better postural control. Administration time of the Mini-BESTest is reported with 15 min [14].

The BBS consists of 14 items; each item is scored out of 4. Individuals need to maintain positions and perform specific balance tasks. Higher scores reflect better postural control. It takes 10–15 min for administration [8]. The BBS revealed good validity, reliability and internal consistency in patients after stroke except for floor and ceiling effects [9].

The TUG measures the time in seconds needed for standing up from a chair, walking three meters straight-ahead, turning around, returning to the chair, and sitting down again. Individuals perform the TUG with their regular walking aids and footwear, but without physical assistance. Healthy controls perform the TUG in 10 s or less [11]. The TUG demonstrated good convergent validity, apart from being a single-task-MI [10].

### Data analysis

Statistical calculation was performed for 50 participants using the statistic software “R v3.5.2”. Descriptive statistics were calculated for gender, age, number of strokes, time and stage after stroke, the use of walking aids and the NIHSS. The MIs were assessed for normal distribution with the calculation of skewness, kurtosis and Q-Q-Plots. Floor and ceiling effects were analysed by calculating percentages for the lowest or highest possible score. The established threshold to identify a floor or ceiling effect is 15% [5]. For hypothesis testing and examining the relationship between the GVMBT and the BBS and the TUG, Spearman’s rank correlation coefficient (r_s_) was used, since the Mini-BESTest and the BBS are ordinal-scaled. The correlation coefficient (r_s_) is interpreted as follows: 0–0.25 little or no relationship; 0.25–0.50 fair relationship; 0.50–0.75 moderate to good relationship and above 0.75 good to excellent relationship [24]. A significant, moderate to high association between MIs would provide evidence of convergent validity [5]. Bland Altman plots were compiled to compare agreement between MIs. Data transformation was necessary, since all tests have different scales. It was performed with the following formulas:

1. BBS transformation = BBS score/56.00*28.00
2. TUG transformation = TUG time/40.08*28.00

**Table:** 

Data transformation was calculated with the highest possible score of the BBS = 56 points and with the longest time required in the TUG = 40.08 s. The highest possible score of the Mini-BESTest is 28 points.	

In order to achieve comparable results, data transformation was calculated with the highest possible score of the BBS and the longest time required in the TUG and then based on the scoring of the Mini-BESTest. Good agreement between two MIs is given if the limits of agreement include the line of equality (0) in the Bland Altman plots [25]. Corresponding scatter plots were calculated with non-transformed data. The internal consistency for the GVMBT was assessed as Cronbach’s Alpha. The minimum value for clinical application is α = 0.90 [5].

## Results

### Participants

A total of 62 individuals after stroke participated in the study. Two persons were excluded because of limited cognitive abilities or cardio respiratory capacity. Ten participants completed pilot-testing and 50 participants were included in statistical analysis. Descriptive characteristics of the participants and scores on all MIs are provided in Table 1. There were no missing data.

**Table 1:** Demographic and clinical characteristics of participants

**Measures** (units)		**n (%)**
**Sum**		50 (100)
**Gender**		
*Female*		16 (32)
*Male*		34 (68)
		**M (SD) [95%CI]**
**Age** (years)		64.58 (13.34) [60.79;68.37]
		**n (%)**
**Number of strokes**		
*One*		37 (74)
*Two*		11 (22)
*Three*		2 (4)
		**M (SD) [95%CI]**
**Time since stroke** (weeks)		24.82 (104.81) [−4.97;54.61]
		**n (%)**
**Stage**		
*Acute*		0 (0)
*Subacute*		28 (56)
*Chronic*		22 (44)
**Walking aids**		
*None*		31 (62)
*Walking stick*		6 (12)
*Walker*		13 (26)
**MIs** (units)	**M (SD) [95%CI]**	**Md (IQR) [Range]**
**NIHSS** (0–42 points)	2.58 (2.09) [1.99;3.17]	2.00 (1.00;4.00) [0.00–7.00]
**Mini-BESTest** (0–28 points)	17.24 (6.71) [15.33;19.15]	17.50 (11.50;23.00) [5.00–28.00]
**BBS** (0–56 points)	44.78 (11.45) [41.53;48.03]	48.50 (39.00;54.75) [13.00–56.00]
**TUG** (seconds)	17.70 (8.73) [15.22;20.18]	16.44 (10.25;23.82) [6.25–40.08]

*n* Number of participants, *M* Mean, *SD* Standard deviation, *95%CI* 95% confidence interval, *Md* Median, *IQR* Interquartile range (25–75%), *MIs* Measurement instruments, *NIHSS* National Institutes of Health Stroke Scale, *Mini-BESTest* Mini-Balance Evaluation Systems Test, *BBS* Berg Balance Scale, *TUG* Timed "Up & Go"

### Translation, adaptation and pretesting

Translation and back translation of the Mini-BESTest were conducted and a committee of investigators discussed and resolved discrepancies. All units were converted to the international metric system. The GVMBT was performed without any problems. Participants reported the Mini-BESTest as comprehensible during performance and relevant concerning their activities of daily living. The average application time of the GVMBT was 16 min.

### Floor and ceiling effects

**Fig. 1:**
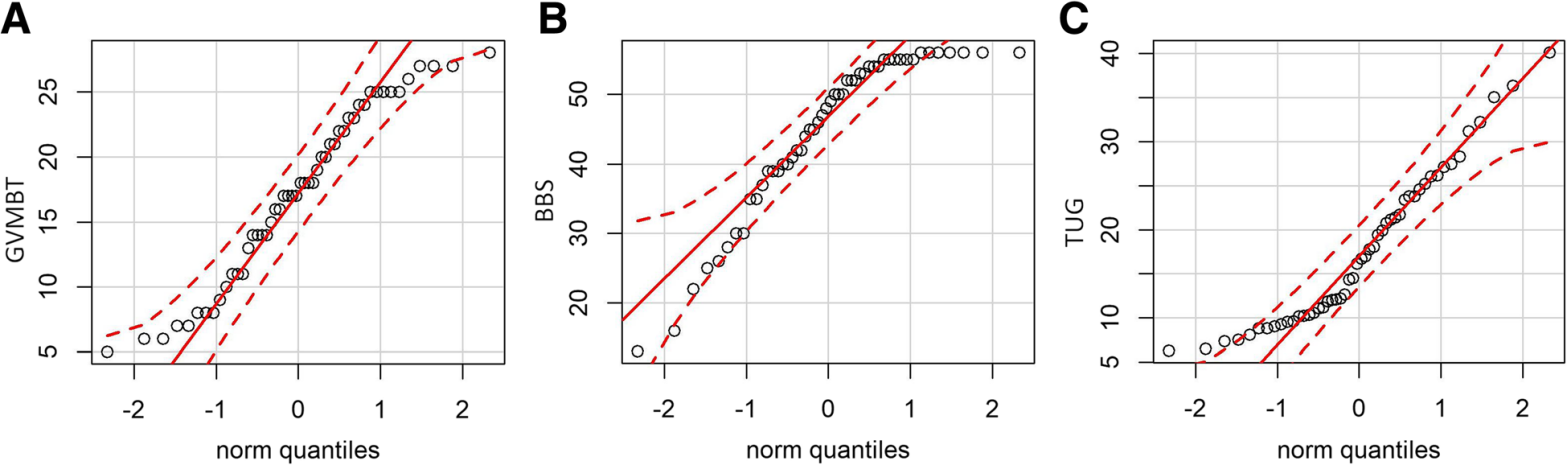
**a**-**c** Q-Q plots of the balance measurement instruments ((**a**) GVMBT; (**b**) BBS; (**c**) TUG) to show score distribution. Q-Q plots examine the score distribution of the **a** GVMBT, the **b** BBS and the **c** TUG by comparing the empirical distribution with a theoretical normal distribution. If the measured values do not scatter around the reference line, it is assumed that there is no normal distribution [24]

No significant floor or ceiling effects were observed for the GVMBT with 2% of the participants reaching the maximum score and none achieving the lowest possible score. In contrast to the GVMBT, the BBS and TUG demonstrated significant deviations of the normal distribution (Table 2 and Fig. 1a-c).

**Table 2:** Comparisons of the GVMBT with the BBS and the TUG: floor and ceiling effects

Measurement instruments (Range of scores)	Skewness [95%CI] ***p***- value	Kurtosis [95%CI] ***p***- value	Floor effect (% participants with lowest possible score)	Ceiling effect (% participants with highest possible score)
**GVMBT** (0–28 points)	−0.19 [− 0.87;0.49] 0.29	−1.10 [−2.46;0.26] 0.06	0	2
**BBS** (0–56 points)	−1.06 [− 1.74;− 0.39] 0.001**	0.40 [− 0.96;1.76] 0.28	0	14
**TUG** (time in seconds)	0.68 [0.01;1.36] 0.02*	-0.39 [−1.74;0.97] 0.29	NA	NA

*GVMBT* German version of the Mini-Balance Evaluation Systems Test, *BBS* Berg Balance Scale, *TUG* Timed "Up & Go", *s* Seconds, *95% CI* 95% Confidence interval, *SE* Standard error, *NA* Not applicable

* p < .05, ** p < .01

### Validity

Significant relationships were found between the GVMBT and the BBS (r_s_ = 0.93, *p* < 0.001) and TUG (r_s_ = − 0.85, *p* < 0.001) (Table 3). These high associations confirm convergent validity of the GVMBT.

**Table 3:** Spearman’s correlations between the GVMBT and the BBS and the TUG

Measurement instruments	Spearman’s rho with the GVMBT
Sum	*n* = 50
**BBS**	0.93***
**TUG**	− 0.85***

The correlation coefficient (r_s_) is interpreted as follows: 0–0.25 little or no relationship; 0.25–0.50 fair relationship; 0.50–0.75 moderate to good relationship and above 0.75 good to excellent relationship [24]

*GVMBT* German version of the Mini-Balance Evaluation Systems Test, *BBS* Berg Balance Scale, *TUG* Timed "Up & Go"

*** p < .001

### Agreement with Bland Altman analysis

**Fig. 2:**
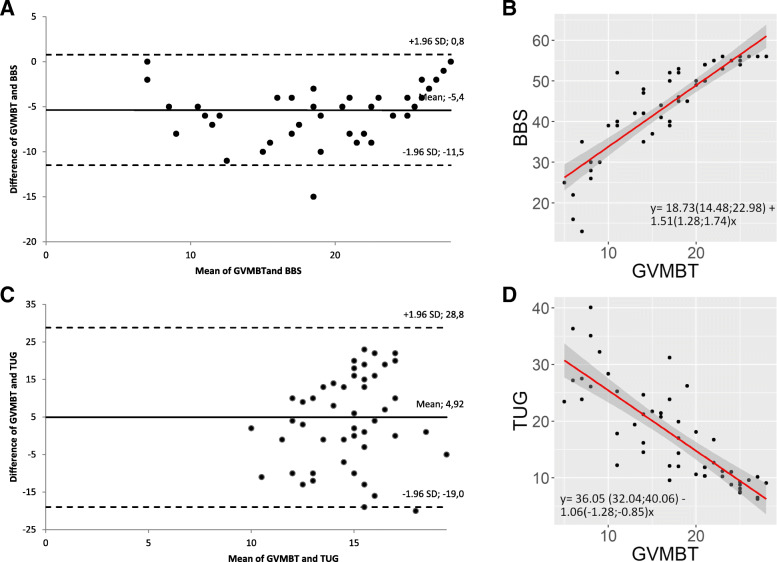
**a**-**d** Bland Altman analysis of the balance measurement instruments to visualize differences between (**a**) GVMBT and the BBS and (**c**) GVMBT and TUG. Bland Altman plots of the balance MIs and corresponding scatter plots. BA plots describe the agreement between two methods: **a** GVMBT and BBS; **c** GVMBT and TUG, where the difference is plotted against the mean. The solid line reveals the bias and the dashed lines represent the upper and lower limits of agreement as bias ± 1.96 standard deviation. Scatter plots show the relationship between the balance MIs: **b** GVMBT and BBS; **d** GVMBT and TUG. The regression equation is expressed as: y = a (95% CI) + b (95% CI) x. The correlation coefficient with its 95%CI between the GVMBT and the BBS is rs = 0.93 (0.88;0.96) and between the GVMBT and the TUG is rs = − 0.85 (− 0.91;-0.75)

In addition, Bland Altman analysis revealed sufficient agreement between all MIs (GVMBT-BBS/TUG). The biases between the GVMBT and the BBS or the TUG were − 5.36 or 4.92, respectively (Table 4). The bias describes the difference between two methods. The comparison between the GVMBT and the BBS demonstrated a smaller variation compared to the GVMBT and the TUG. Both comparisons included the line of equality, which shows that there is no significant difference between the MIs. However, Fig. 2a+c visualized that the line of equality was more centred between the GVMBT and the TUG. The corresponding scatter plots revealed the association between the compared MIs (Fig. 2b+d).

**Table 4:** Bland Altman plots statistics

	Bias [95%CI]	ULOA [95%CI]	LLOA [95%CI]	SE for LOA	Variation	SE of bias	Min. of mean	Max. of mean
**GVMBT and BBS**	−5.36 [− 6.25; − 4.47]	0.77 [− 0.76; 2.30]	− 11.49 [− 13.02; − 9.96]	0.76	3.13	0.44	7.00	28.00
**GVMBT and TUG**	4.92 [1.46; 8.38]	28.81 [22.85; 34.77]	−18.97 [− 24.93; − 13.01]	2.97	12.19	1.72	10.00	19.50

*GVMBT* German version of the Mini-Balance Evaluation Systems Test, *BBS* Berg Balance Scale, *TUG* Timed "Up & Go", *CI* Confidence interval, *ULOA* Upper limit of agreement, *LLOA* Lower limit of agreement, *SE* Standard error, *LOA* Limits of agreement, *SD* Standard deviation, *MI* Measurement instrument, *Min.* Minimum, *Max.* Maximum; formula for LOA = d ± 1.96 SD; bias = mean difference; variation = SD of mean difference; SE formula for a) LOA= $\sqrt{3S{D}^2/n}$; b) bias= $\sqrt{S{D}^2/n}$ [23]

### Internal consistency

The internal consistency for all 14 items of the GVMBT was excellent (Cronbach’s *α = 0.90, 95% CI of 0.87–0.94).*

## Discussion

This is the first study to translate, cross-culturally adapt the Mini-BESTest into the German language and to examine validity of the German version of the Mini-BESTest with individuals after stroke. The GVMBT was comprehensible and well accepted by participants and physiotherapists. Clinical utility was supported by the average duration of the GVMBT of 16 min. Our results show that the GVMBT has excellent validity, internal consistency and demonstrate sufficient agreement in the Bland Altman analysis, as well as no floor and no ceiling effects compared to commonly used balance MIs. These findings are in accordance with our hypotheses. The study sample in a validation study should reflect the population of interest [5]. To cover a heterogeneous population of people with mild to moderate stroke, we included participants with various severities at two different rehabilitation centres. We succeeded to recruit a heterogeneous sample, as the total scores for the Mini-BESTest ranged from five to 28 points with patients having one to three strokes and using walking aids or being free walkers. Correspondingly, the scores for the Mini-BESTest showed a considerable variability (Table 1). Thus, our results can likely be generalised to a group of mild to moderate affected persons after stroke.

### Translation and acceptability

Linguistic and content-related discrepancies were clarified in correspondence with the original developer [14] and an interdisciplinary committee of experts. The GVMBT was comprehensible and well accepted by participants and physiotherapists, which is in accordance with other translational studies of the Mini-BESTest [20–22]. Consistently, the application of the German version was comparable to the original version.

### Floor and ceiling effects

The GVMBT was significantly less skewed and displayed the least ceiling effect compared to the BBS and TUG. In contrast, the BBS and the TUG demonstrated significant skewness. As expected, the BBS almost revealed a ceiling effect of 14% in our participants reaching the highest possible score, which is close to the established threshold of 15% [5]. A recent review of the psychometric properties of the Mini-BESTest reported a ceiling effect ranging from 0.9 to 4.3% in various populations [16], which is similar to our results, showing a ceiling effect of 2%. Tsang and colleagues [26] determined a skewness of 2.69 in the BBS and 1.69 in the TUG, which are similarly distributed to our findings. Furthermore, the authors reported a ceiling effect of 32.1% for the BBS in persons with chronic stroke [26]. Our results revealed no floor effects in any MI, since the inclusion criteria assume a walking ability. A previous review reported a floor effect for the Mini-BESTest in a small group of individuals with strong impairments [16]. Additionally, the Mini-BESTest covers, with its four subcategories, different aspects of postural control and includes more challenging tasks, in contrast to other MIs. The BBS and TUG are more limited and do not include the comprehensive examination of anticipatory postural control, reactive postural control, sensory orientation and dynamic gait aspects, including dual-task [14]. As a result, the subcategories may have improved the discrimination between participants in the GVMBT, too.

### Validity and agreement between measurement instruments

The excellent construct validity of the GVMBT is reflected by very high correlations between the GVMBT and the BBS or TUG. The correlations strengthen the assumption that the MIs assess the construct postural control and thus support the construct validity. Our results are in agreement with previous validity studies on the original [14, 16], Greek [22] and Swedish [20] version and in individuals after stroke [26], persons with balance disorders, Parkinson’s disease, spinal cord injury [21] and adults aged 50 years and older [16]. Other studies also investigated the construct validity of the Mini-BESTest with different subjective outcomes like fall efficacy and health-related quality of life. For instance, five papers assessed the concurrent validity between the Mini-BESTest and measures of fall efficacy in different populations [20, 27–30]. A low correlation with the fall efficacy scale (*r* = 0.26) [20] was reported and moderate correlations with the activities-specific balance confidence scale (*r* between 0.53 and 0.66, *p* = 0.52) [27–30]. The Mini-BESTest may not reflect the performance in everyday life in detail and may therefore not be transferable to the estimated fear of falling. However, the Mini-BESTest may indicate, if there is an increased fear of falling. To date, only one study investigated the relation of health-related quality of life (Quality of Life Questionnaire-C30) and postural control (Mini-BESTest) in patients with cancer. It showed positive correlations between the Mini-BESTest and all domains of the Quality of Life Questionnaire-C30 [31]. Further studies, especially with other populations are needed.

However, since high correlations do not automatically imply an agreement between MIs, Bland Altman analysis was applied [25]. Our results suggest that the comparisons of the GVMBT with the BBS and TUG expose no significant bias, since the lines of equality were included in the limits of agreement and the mean differences were relatively low. Additionally, the standard deviation was smaller for the comparison of the GVMBT and BBS. In this study the GVMBT showed no over- or underestimation of balance performance.

### Internal consistency

Internal consistency of the GVMBT with a Cronbach’s *α* of 0.90 was considered as excellent. Our values are in agreement with those previously reported in different populations with Cronbach’s α ranging from 0.89–0.96 [16]. Moreover, it is comparable to other translated versions as for instance the Greek version with Cronbach’s α of 0.95 [22].

### Limitations

Although the present study has shown that the GVMBT is valid and comprehensible, it has some limitations. Our findings are only generalizable to individuals in the subacute or chronic stages of stroke recovery and the sample size was too small for further analysis such as item-response theory or Rasch analysis. Although there are many different MIs to assess postural control such as the Dynamic Gait Index [6, 13], we decided to administer the most commonly used balance MIs in stroke rehabilitation and research to ensure feasibility and to avoid patient fatigue.

## Conclusion

In conclusion, the instructions and scoring descriptions of the GVMBT are equivalent to those of the original version. The GVMBT has excellent validity and internal consistency, which is in accordance to previous studies investigating these properties of the Mini-BESTest in various populations and other languages. Considering these studies, our findings and the four specific subcategories of the Mini-BESTest, the GVMBT can be recommended for the use in clinical practice and research. Due to the score distribution within the BBS and the Mini-BESTest and the difficulty of the items, the GVMBT might be more appropriate for less impaired individuals. Since the Mini-BESTest contains four different subcategories reflecting almost all aspects of postural control and its short application time, the GVMBT is informative and conveniently for clinical practice. Further studies should focus on other psychometric properties of the GVMBT in larger populations with different functional limitations. Additionally, average values of the GVMBT could serve as an orientation regarding the necessity of walking aids and should be further analyzed.

## Data Availability

The datasets used and analysed during the current study are not publicly available due to the declaration of consent, which does not allow the transfer of anonymous data.

## Abbreviations

BBS: Berg Balance Scale
BESTest: Balance Evaluation Systems Test
GVMBT: German version of the Mini-BESTest
MI: Measurement instrument
NIHSS: National Institutes of Health Stroke Scale
r_s_: Spearman’s rank correlation coefficient
TUG: Timed “Up & Go”
